# Case Report: Tezepelumab as rescue therapy in near-fatal asthma requiring extracorporeal membrane oxygenation

**DOI:** 10.3389/fimmu.2026.1722963

**Published:** 2026-02-13

**Authors:** Gani Oruqaj, Johannes Bewersdorff, Detlef Litzlbauer, Matthias Wolff, Michael Sander, Werner Seeger, Friedrich Grimminger, Matthias Hecker, Khodr Tello, István Vadász

**Affiliations:** 1 Department of Internal Medicine, Justus Liebig University, Giessen, Germany; 2 Universities of Giessen and Marburg Lung Center, German Center for Lung Research, Giessen, Germany; 3 Interdisciplinary Extracorporeal Membrane Oxygenation (ECMO) Center, Interdisciplinary Center for Intensive Care Medicine, University Hospital Giessen, Giessen, Germany; 4 Department of Diagnostic and Interventional Radiology, University Hospital Giessen, Giessen, Germany; 5 Department of Anesthesiology, Intensive Care Medicine and Pain Therapy, Justus Liebig University Giessen, Giessen, Germany; 6 Insitute for Lung Health, Giessen, Germany; 7 Cardio-Pulmonary Institute, Giessen, Germany

**Keywords:** status asthmaticus, Tezepelumab, TSLP, ICU, ECMO

## Abstract

**Background:**

Tezepelumab, an anti-thymic stromal lymphopoietin (TSLP) monoclonal antibody, reduces exacerbations across asthma phenotypes, but its role in status asthmaticus and near-fatal exacerbations requiring veno-venous extracorporeal membrane oxygenation (VV-ECMO) is unclear.

**Methods and Results:**

We report three patients (17, 22, 57 years) with therapy-refractory hypercapnic respiratory failure initiated on VV-ECMO who received tezepelumab 210 mg within 24 h. Tidal volume and minute ventilation increased within 24–72 h, permitting decannulation by days 12–15 and ventilator weaning by days 17–28. Two patients had elevated IgE; one had normal blood eosinophils/IgE. No immediate drug-related adverse events occurred. Follow-up demonstrated lung-function recovery; one patient required escalation of maintenance therapy for persistent symptoms.

**Conclusion:**

In this small series, adjunctive tezepelumab during VV-ECMO–supported status asthmaticus appeared safe and potentially beneficial adjunctive therapy during near-fatal asthma requiring VV-ECMO. Randomized controlled studies are needed to determine the impact of TSLP inhibition on recovery time, ventilation duration, and mortality in this setting.

## Graphical Abstract

**Figure:**
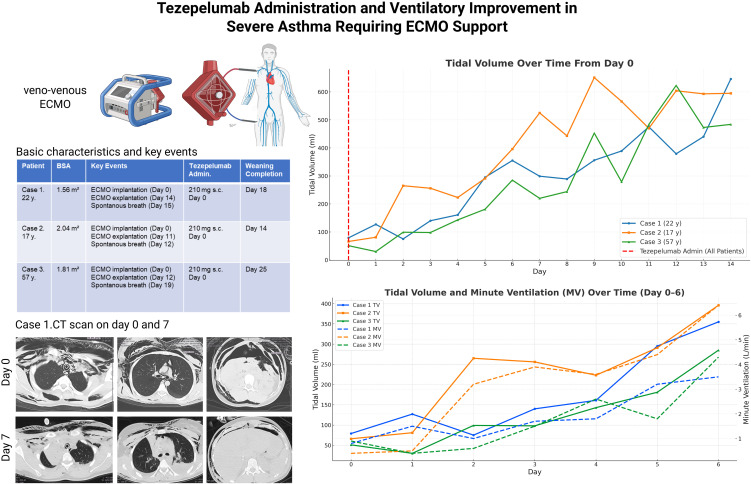
Graphical summary of three patients with life-threatening asthma requiring veno-venous ECMO who received a single dose of tezepelumab. Clinical timelines and key events are shown alongside imaging from Case 1 demonstrating resolution of barotrauma between Day 0 and Day 7. Tidal volume increased within the first 24–48 hours after tezepelumab across all cases, with corresponding improvements in minute ventilation during Days 0–6. These observations suggest a potential early physiological benefit of TSLP blockade during severe, refractory asthma exacerbations.

## Introduction

Monoclonal antibodies have transformed severe asthma care. Thymic stromal lymphopoietin (TSLP) is an epithelial-derived cytokine released early in response to airway injury and allergen exposure, functioning as an upstream driver of both type 2 and non–type 2 inflammatory pathways (1). Tezepelumab, a human monoclonal antibody against TSLP, targets the inflammatory cascade at its point of initiation, providing broad efficacy across asthma phenotypes, reduces exacerbations and improves FEV_1_, symptom control, and quality of life independent of blood eosinophil levels (1–3).

Whether biologics can modify the course of ongoing life-threatening exacerbations is unclear. No monoclonal antibody has been prospectively tested for reversing airflow obstruction during status asthmaticus, shortening mechanical ventilation, or improving outcomes with extracorporeal support. In selected cases of refractory hypercapnic respiratory failure, veno-venous extracorporeal membrane oxygenation (VV-ECMO) enables lung rest and extracorporeal CO_2_ removal and can be life-saving, with contemporary series reporting low mortality in severe asthma (4). Additional adjuncts—volatile anesthetics (sevoflurane), nebulized magnesium, ketamine—may help but are often limited early when tidal volumes are extremely low (5). Case reports describe biologic use (omalizumab, benralizumab) in mechanically ventilated status asthmaticus, and a recent report documented tezepelumab during ECMO with apparent improvement (6–8).

Despite these isolated reports, systematic data on biologic therapy in the acute ICU setting are lacking. We report three consecutive patients with refractory status asthmaticus requiring invasive ventilation and VV-ECMO who received adjunctive tezepelumab early after cannulation. We describe feasibility, short-term physiologic response (tidal volume and minute ventilation), weaning timelines, and follow-up, and discuss implications and research priorities for targeted biologic intervention during near-fatal asthma.

## Cases

### Case 1

**Figure 1:**
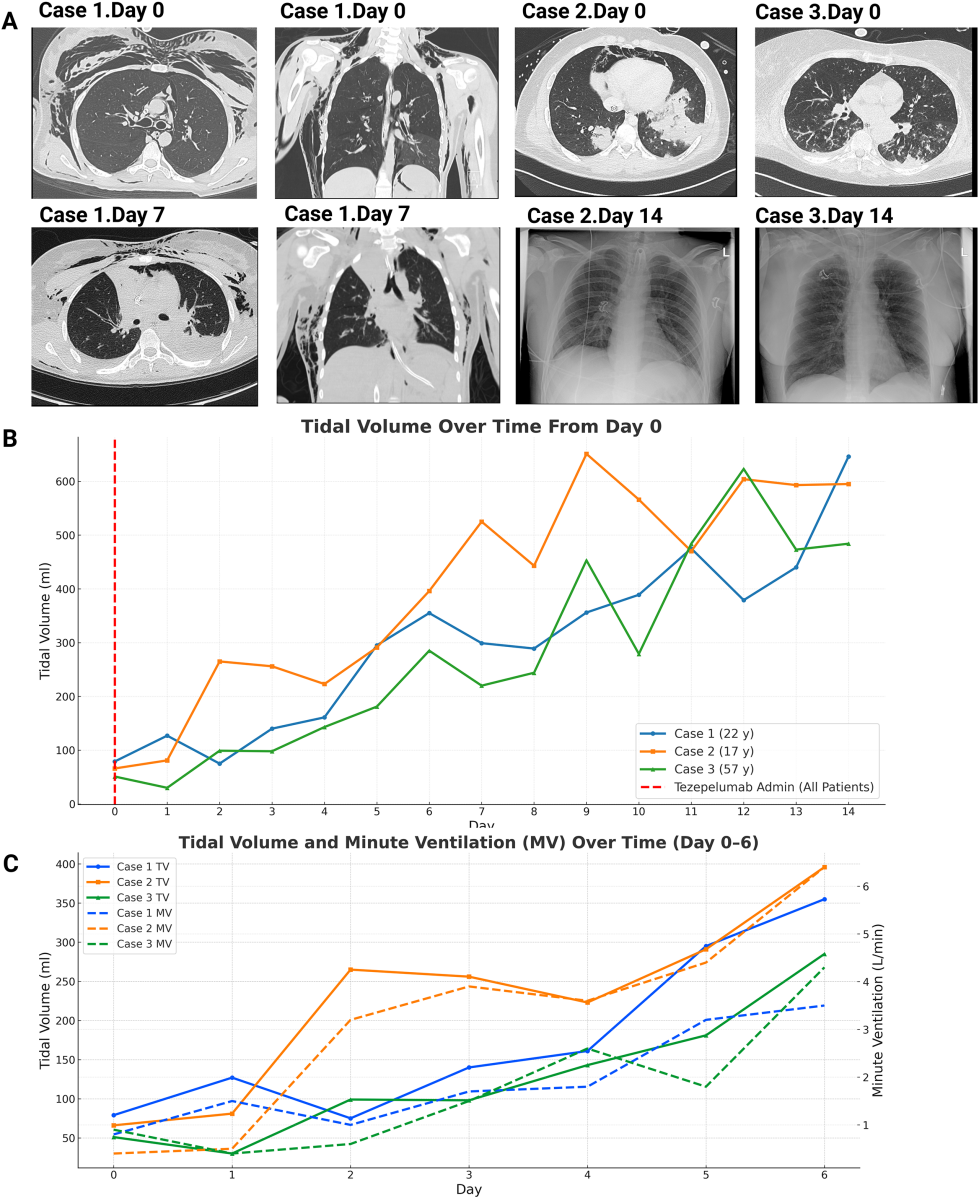
Radiographic evaluation, evolution and ventilation parameters in patients with status asthmaticus treated with vv-ECMO and tezepelumab. **(A)** CT thorax images of Case 1 (22 years) on Day 0 (top row) and Day 7 (bottom row) following ECMO initiation and tezepelumab administration. On Day 0, extensive subcutaneous emphysema, pneumomediastinum, and pneumoperitoneum are evident, reflecting severe barotrauma. By Day 7, there is marked improvement with resolution of soft tissue emphysema, pneumomediastinum, and intra-abdominal free air. Radiographic findings in Case 2 (17 years) and Case 3 (57 years). Case 2 shows CT evidence of bilateral ground-glass opacities, pneumomediastinum, and consolidations on Day 0, with follow-up chest radiographs demonstrating improved lung inflation and resolution of mediastinal air by Day 14. Case 3 presents with diffuse bilateral infiltrates and subpleural consolidations at baseline, with serial radiographs showing progressive clearing and clinical improvement following ECMO support and tezepelumab therapy. **(B)** Ventilation parameters following tezepelumab administration. Daily tidal volume (TV, mL) trends in all three patients demonstrated consistent improvement over 14 days (solid lines), with tezepelumab administered on Day 0 (red dashed line). **(C)** Corresponding changes in TV and minute ventilation (MV, L/min) during the first 7 days are also shown, with solid lines representing TV and dashed lines representing MV. A progressive rise in both parameters was observed, particularly between Days 2 and 6, indicating improved ventilation and gas exchange during ECMO and biological therapy. TV, tidal volume; MV, minute ventilation; ECMO, extracorporealmembrane oxygenation.

A 22-year-old woman with allergic asthma, treated with tiotropium bromide/olodaterol hydrochloride and as-needed salbutamol, was admitted to an external hospital with a severe exacerbation. Despite intubation, ventilation remained inadequate (PaCO_2_ 90 mmHg), prompting emergency veno-venous extracorporeal membrane oxygenation (VV-ECMO) cannulation by our mobile team and transfer to our ICU. Initial imaging demonstrated extensive subcutaneous emphysema and parenchymal tears, consistent with the Macklin phenomenon (**Figure 1A**). On the day of ECMO initiation, the patient received a single subcutaneous dose of tezepelumab (210 mg), 6 hours after ECMO cannulation. On admission, the patient had a markedly elevated total IgE level of 1,705.6 IU/mL (normal range <100 IU/mL). Blood eosinophils were 150/µl, and a repeat measurement two months later remained low 150/µl, with a historical value of 700/µl recorded in 2016. A CT scan performed on day 0 showed right upper lobe and left lower lobe atelectasis, and a bronchoscopy was conducted on day 1 and day 4 due to viscous mucus plugs. A tracheostomy was placed on day 10 as part of prolonged ventilatory support. Adjunctive therapy included intravenous prednisolone 40 mg daily for 3 weeks, magnesium sulfate, and reproterol, together with inhaled budesonide and salbutamol. Sedation was maintained with esketamine and midazolam, later transitioned to sevoflurane. During ECMO, ultra-protective lung ventilation was applied. The patient remained on ECMO for 15 days and was fully weaned from invasive ventilation by Day 20, demonstrating gradual clinical improvement with a noticeable increase in tidal volume beginning approximately 72 hours after tezepelumab administration. At three-month follow-up, pulmonary function testing demonstrated good recovery, with normalization of vital capacity and only mild airflow limitation, FEV_1_/FVC ratio of 98%, and FeNO was 11 ppb. **Table 1** summarizes the patient’s baseline characteristics, comorbidities, clinical course, lung function, and serum markers.

**Table 1:** Summary of patient characteristics, ECMO and clinical course, comorbidities, biomarkers and follow-up.

Parameter	Case 1 22 y, female	Case 2 17 y, male	Case 3 57 y, female
Phenotype and Body Surface Area (BSA)	Allergic 1.56 m²	Non-allergic 2.04 m²	Allergic 1.81 m²
Key Events	ECMO Day 0; explant Day 14; spontaneous breath Day 15	ECMO Day 0; explant Day 11; spontaneous breath Day 12	ECMO Day 0; explant Day 12; spontaneous breath Day 19
ECMO Duration	0–14 days	0–11 days	0–12 days
Tezepelumab Administration	210 mg s.c. Day 0	210 mg s.c. Day 0	210 mg s.c. Day 0
Tracheotomy	Day 10	Day 6	Day 6
Weaning Completion	Day 18	Day 14	Day 25
Comorbidities	Infection-triggered exacerbations; von Willebrand–Jürgens syndrome; active tobacco use	No known comorbidities nonsmoker	Active tobacco use (50 pack-years); depression; hypothyroidism; arterial hypertension; hypercholesterolaemia
Pre-ECMO ABG	ECMO initiated externally; no pre-cannulation ABG	PaCO_2_ unmeasurable; severe ventilatory failure	pH 6.96, PaCO_2_ 147 mmHg, pO_2_ 76 mmHg, BE –5.8 mmol/L, Hb 121 g/L
Blood Eosinophils	150/µL on admission	230/µL on admission	0/µL on admission
Serum IgE	1,705 IU/mL; 960 IU/mL at 3 mo	26 IU/mL	423 IU/mL
Imaging Summary	CT Day 0: Barotrauma; CT Day 7: Marked resolution	CT Day 0: bilateral opacities, pneumomediastinum x-ray day 14: near-complete resolution	CT Day 2: Tree−in−bud consolidations; X-ray day 14: near−complete resolution
Bronchoscopy	Day 1 and 4: mucus plugs	Day 2, 4, 5: purulent secretions	Day 1: Inflamed mucosa; thick secretions
Follow-up Lung Function	3 mo: FEV_1_/FVC 80%, VC 86%, RV 77%, R_e_ff 88%, FeNO 11 ppb	1 mo: FEV_1_/FVC 78%, VC 103%, RV 166%; 4 mo: FEV_1_ 105%, VC 118%, RV 95%	4 mo: FEV_1_/FVC 56%, VC 104%; 8 mo: FEV_1_ 54%, VC 91%, RV 209%

Summary of patient characteristics, clinical course, comorbidities, pre-ECMO arterial blood gases, serum IgE levels, and lung function follow-up.

This table provides an overview of three patients with status asthmaticus who required VV-ECMO support and received adjunctive tezepelumab therapy. Demographic data (body surface area), comorbidities, and the sequence of key clinical events—including ECMO initiation, tracheotomy, and ventilation weaning—are documented. All patients received 210 mg tezepelumab subcutaneously within 24 hours of ECMO therapy.

Pre-ECMO arterial blood gas (ABG) parameters are shown where available. Serum IgE levels were measured at baseline and during follow-up. Lung function tests were performed at defined intervals, including FEV_1_, vital capacity (VC), residual volume (RV), and effective airway resistance (R_e_ff), with fractional exhaled nitric oxide (FeNO) where available. Lung function values are reported as percent predicted unless stated otherwise. Missing data are denoted by “—”.

ABG, arterial blood gas; BSA, body surface area; BE, base excess; ECMO, extracorporeal membrane oxygenation; FeNO, fractional exhaled nitric oxide; Hb, hemoglobin; MV, minute ventilation; pred, predicted; R_e_ff, effective airway resistance; s.c., subcutaneous; VC, vital capacity, Trach: Tracheostomy.

### Case 2

A 17-year-old male with asthma diagnosed in early childhood, previously hospitalized at ages 11 and 14 but managed only with as-needed salbutamol, presented to an outside hospital with severe dyspnea limiting speech. He was intubated for respiratory exhaustion; however, minute ventilation remained <1 L/min and PaCO_2_ was unmeasurable (>150 mmHg), prompting emergency veno-venous extracorporeal membrane oxygenation (VV-ECMO) and transfer to our ICU (**Figure 1**, **Table 1**). Initial therapy included inhaled and intravenous bronchodilators, systemic prednisolone 50 mg daily for 3 weeks, and magnesium sulfate. Adjunctive therapies included sevoflurane beginning on day 2, inhaled budesonide, esketamine, and reproterol starting on day 0. A single subcutaneous dose of tezepelumab (210 mg) was administered on ICU day 1, 18 hours after ECMO initiation. On admission, the patient’s total IgE level was 26 IU/mL (normal <100 IU/mL), and blood eosinophils measured 230/µl on day 5. The patient was intubated externally and transferred after receiving 500 mg intravenous prednisolone from an emergency physician. A tracheostomy was performed on day 6 due to anticipated prolonged ventilation. Bronchoscopies performed on days 2, 4, and 5 revealed purulent secretions consistent with severe airway obstruction. An initial CT scan showed bilateral ground glass opacities, pneumomediastinum and consolidation on day 0, with a follow-up chest radiograph on day 14 demonstrating improved lung inflation and resolution of the pneumomediastinum. IgE and eosinophil levels were within the normal range, consistent with a low blood eosinophil and IgE phenotype. VV-ECMO support was maintained for 12 days, with tracheostomy performed on day 7. The patient was successfully weaned from mechanical ventilation on day 17 and demonstrated favorable recovery thereafter. Baseline spirometry showed an FEV_1_/FVC of 97% at follow-up measurement on day 26. Baseline data, clinical events, and follow-up are summarized in **Table 1**.

### Case 3

A 57-year-old woman with asthma–COPD overlap syndrome and an extensive smoking history (50 pack-years) presented to an external hospital with acute hypercapnic respiratory failure. Her maintenance therapy included montelukast and inhaled fluticasone furoate/umeclidinium/vilanterol. Despite invasive ventilation, severe hypercapnia persisted (PaCO_2_ 107 mmHg), and she was transferred to our ICU the following day. Tidal volumes remained critically low (~50 mL) despite appropriate ventilator settings (peak inspiratory pressure 26 cmH_2_O, PEEP 12 cmH_2_O), prompting initiation of VV-ECMO. On ICU day 0, she received tezepelumab (210 mg subcutaneously) as adjunctive therapy, 20 hours after ECMO initiation (**Table 1**, **Figure 1**). At presentation, the patient had a markedly elevated total IgE level of 423.1 IU/mL (normal range<100 IU/mL), while blood eosinophils were 0/µl on day 0. A tracheostomy was performed on day 6. A CT scan on day 2 demonstrated multiple, partially confluent fine nodular peribronchiovascular opacities consistent with a tree-in-bud pattern, along with bronchial wall thickening and large consolidations in both posterior lower lobes, more pronounced on the left. Initial bronchoscopy showed erythematous, swollen, and contact-vulnerable mucosa in the main and segmental bronchi bilaterally, with thick, blood-tinged and tenacious secretions requiring prolonged suctioning. Follow-up chest radiograph on day 14 showed near-complete resolution of lung inflammation. Sedation was maintained with esketamine and midazolam. Additional therapy included systemic corticosteroids 50 mg daily for 3 weeks, inhaled budesonide, ipratropium bromide/fenoterol, and intravenous magnesium. VV-ECMO was explanted after 12 days. Spontaneous breathing was established on day 19, and complete weaning was achieved on day 28. Spirometry performed four months after discharge showed an FEV_1_/FVC ratio of 61%, and FeNO was not measurable during the acute phase. Clinical characteristics and outcomes are summarized in **Table 1**.

## Discussion

Status asthmaticus requiring invasive mechanical ventilation and extracorporeal life support is a life-threatening condition with substantial morbidity and cost, particularly when bronchoconstriction proves refractory to conventional therapy. Although biologics such as tezepelumab have reshaped maintenance management of severe asthma, their role in the ICU during ongoing, near-fatal exacerbations remains largely unexplored. This case series provides preliminary clinical insight into adjunctive tezepelumab in three patients with status asthmaticus supported with VV-ECMO.

TSLP, a key upstream cytokine that amplifies both type 2 (T2) and non-T2 inflammation. In randomized trials, tezepelumab reduced exacerbations and improved lung function across phenotypes, including in patients without elevated blood eosinophils or IgE (3). In our series, tezepelumab (210 mg s.c.) was administered within 24 hours of ECMO cannulation. All patients showed early stabilization with rising tidal volumes and minute ventilation over 24–72 hours, enabling ECMO decannulation by days 12–15 and ventilator weaning by days 17–28. Notably, the youngest patient exhibited a favorable response despite no elevated blood eosinophils and IgE, supporting potential utility beyond classic T2-high exacerbations. These observations align with a recent report of tezepelumab during ECMO that described rapid improvement in airway mechanics (8). Several mechanisms could underlie the observed trajectory. By attenuating upstream epithelial-immune signaling, TSLP blockade may reduce mucosal edema, mucus hypersecretion, and steroid-refractory inflammation, thereby lowering airway resistance (3). Recent case series describe rapid clinical improvement after benralizumab administration in patients with near-fatal eosinophilic asthma who remained refractory to maximal supportive therapy yet did not require ECMO support, underscoring the central role of eosinophils in those phenotypes (9). In contrast, the patients in our report lacked substantial eosinophilia, suggesting that tezepelumab as an upstream alarmin blocker may provide therapeutic benefit in a broader range of inflammatory profiles during severe exacerbations. Early physiologic gains in ventilation mechanics can facilitate safer ventilator settings and, in some cases, enable the use of adjuncts for sedation therapy such as volatile anesthetics once tidal volumes become adequate.

VV-ECMO remains a critical salvage option for refractory hypercapnic respiratory failure in status asthmaticus, offering lung rest and extracorporeal CO_2_ removal with contemporary mortality rates around 6–10% and generally favorable functional outcomes (4). Our patients, consistent with these reports, all survived, further supporting the role of ECMO as an effective bridge to recovery in this setting, as well as highlighting the role of mobile ECMO teams in providing fast therapy on-site and ensuring transfer to specialized referral clinics.

Low-flow and ultra-low-flow extracorporeal CO_2_ removal (ECCO_2_R) methodologies are emerging as minimally invasive strategies for selected patients, offering earlier intervention for hypercapnia with potentially fewer complications (10). A case series by Fox et al. showed an 88% survival rate in patients with refractory hypercapnia treated with ECCO_2_R (11). Such approaches may be integrated as alternatives to ECMO in select cases (12). This study has limitations: small sample size, lack of controls, and concurrent therapies (systemic corticosteroids, bronchodilators, magnesium, sedation) confound attribution. Furthermore, in case 1 and 2, the absence of guideline-recommended inhaled corticosteroid (ICS) therapy despite a history of allergic or early-onset asthma likely contributed to disease instability; this undertreatment represents a critical deviation from current GINA recommendations and underscores the importance of adequate controller therapy to prevent progression to near-fatal asthma.

Despite these constraints, the convergence of early physiologic improvement, feasibility of administration, and absence of immediate drug-related adverse events supports additional investigation. Future studies should evaluate optimal timing and dosing of tezepelumab in the ICU, characterize pharmacokinetics/pharmacodynamics under ECMO, and assess biomarker-defined subgroups (T2-high vs non-T2). Clinically meaningful endpoints include ventilator-free days, ECMO duration, ICU length of stay, and mortality, alongside safety outcomes. Randomized or pragmatic adaptive designs with mechanistic sampling (e.g., airway and plasma cytokines) would help determine whether TSLP inhibition can accelerate recovery in near-fatal asthma.

## Data Availability

The original contributions presented in the study are included in the article/supplementary material. Further inquiries can be directed to the corresponding author.
